# Polysaccharides from New Zealand Native Plants: A Review of Their Structure, Properties, and Potential Applications

**DOI:** 10.3390/plants8060163

**Published:** 2019-06-09

**Authors:** Susan M. Carnachan, Tracey J. Bell, Simon F. R. Hinkley, Ian M. Sims

**Affiliations:** Ferrier Research Institute, Victoria University of Wellington, 69 Gracefield Road, Lower Hutt 5040, New Zealand; Susie.Carnachan@vuw.ac.nz (S.M.C.); Tracey.Bell@vuw.ac.nz (T.J.B.); Simon.Hinkley@vuw.ac.nz (S.F.R.H.)

**Keywords:** novel polysaccharide, characterization, rheology, New Zealand, applications

## Abstract

Water-soluble, non-starch polysaccharides from plants are used commercially in a wide range of food and non-food applications. The increasing range of applications for natural polysaccharides means that there is growing demand for plant-derived polysaccharides with different functionalities. The geographical isolation of New Zealand and its unique flora presents opportunities to discover new polysaccharides with novel properties for a range of applications. This review brings together data published since the year 2000 on the composition and structure of exudate gums, mucilages, and storage polysaccharides extracted from New Zealand endemic land plants. The structures and properties of these polysaccharides are compared with the structures of similar polysaccharides from other plants. The current commercial use of these polysaccharides is reviewed and their potential for further exploitation discussed.

## 1. Introduction

The geological isolation of New Zealand has resulted in its distinctive flora and fauna, with about 80% of the more than 2300 species of vascular plants being endemic. This unique biodiversity presents the possibility of discovering new compounds with novel biological or chemical properties and, correspondingly, novel applications. Historically, relatively few native plants were used as food sources by the Māori, but a wide range of plants were used for medicinal purposes [1]. Recently, of almost 400 compounds with medicinal properties isolated from New Zealand plants, about 10% were considered as having potential as lead drug candidates or therapeutics [2]. Earlier, a survey of extracts from 344 native New Zealand plants found widespread cytotoxic and anti-bacterial activity [3]. Polysaccharides are an abundant group of polymers with many industrial applications. The unique environments in New Zealand and its biodiversity present opportunities for the discovery of new plant polysaccharides with novel properties and functions. Among the many New Zealand medicinal plants shown in the book by Brooker, Cambie, and Cooper [1], only a few are mentioned to contain gum or mucilage as an active component; the gum of some of these plants (e.g., red pine, rimu) are probably terpenoid resins and not polysaccharide gums. 

The potential for novel polysaccharides from New Zealand seaweeds has been the subject of considerable research effort, leading to the discovery of novel high melting-point agars with particular methyl-ether substituents [4] and new sources of carrageenans with potential dairy applications [5,6]. Polysaccharides from New Zealand fungi have been isolated, characterized, and analyzed for their antibacterial and antioxidant activities [7]. The same group isolated a novel, uronic acid-rich polysaccharide from the mushroom (*Iliodiction cibarium*) that was historically consumed by Māori [8].

In this review, we summarize the literature since the year 2000 on the structure and biological and functional properties of novel polysaccharides extracted from New Zealand native land plants. We discuss their potential utilization in relation to commercially available polysaccharides, and polysaccharides with similar compositions and structures. The review is primarily limited to polysaccharides that have been extensively characterized, although the potential for other plants to yield novel polysaccharide structures is discussed.

## 2. Exudate Gums and Mucilages from NZ Plants

Many plants produce exudate gums or mucilages; gums are usually produced in response to wounding or some other abiotic stress, such as disease, while mucilages are produced as part of the normal metabolism of the plant [9]. These water-soluble, non-starch polysaccharides are used commercially for their ability to alter the physicochemical properties of aqueous solutions and have a wide range of food and non-food applications [10]. Currently, gum arabic is the most widely used exudate gum, and other exudate gums, such as tragacanth, karaya, and ghatti, which were previously important, are used in only small amounts today [10,11]. Polysaccharide mucilages, including those from okra (*Abelmoschus esculentus*), and other members of the family Malvaceae, and from seeds, such as chia (*Salvia hispanica*), basil (*Ocimum basilicum*) and *Plantago* species, are not produced commercially, but are being actively researched [9,10].

### 2.1. Puka (*Meryta sinclairii*)

Puka is a member of the family Araliaceae, which comprises 28 species found within the tropical and subtropical Pacific. Puka is native to Three Kings Island off the northern tip of New Zealand, but it is commonly grown as an ornamental plant throughout the north island [12]. The trunk exudes a clear gum when wounded, which dries to a hard, glass-like material containing about 70% solids. The purified gum is completely precipitated using β-glucosyl Yariv reagent, a synthetic phenyl glycoside that specifically binds to and precipitates plant arabinogalactan-proteins (AGPs) [13]. 

**Figure 1:**
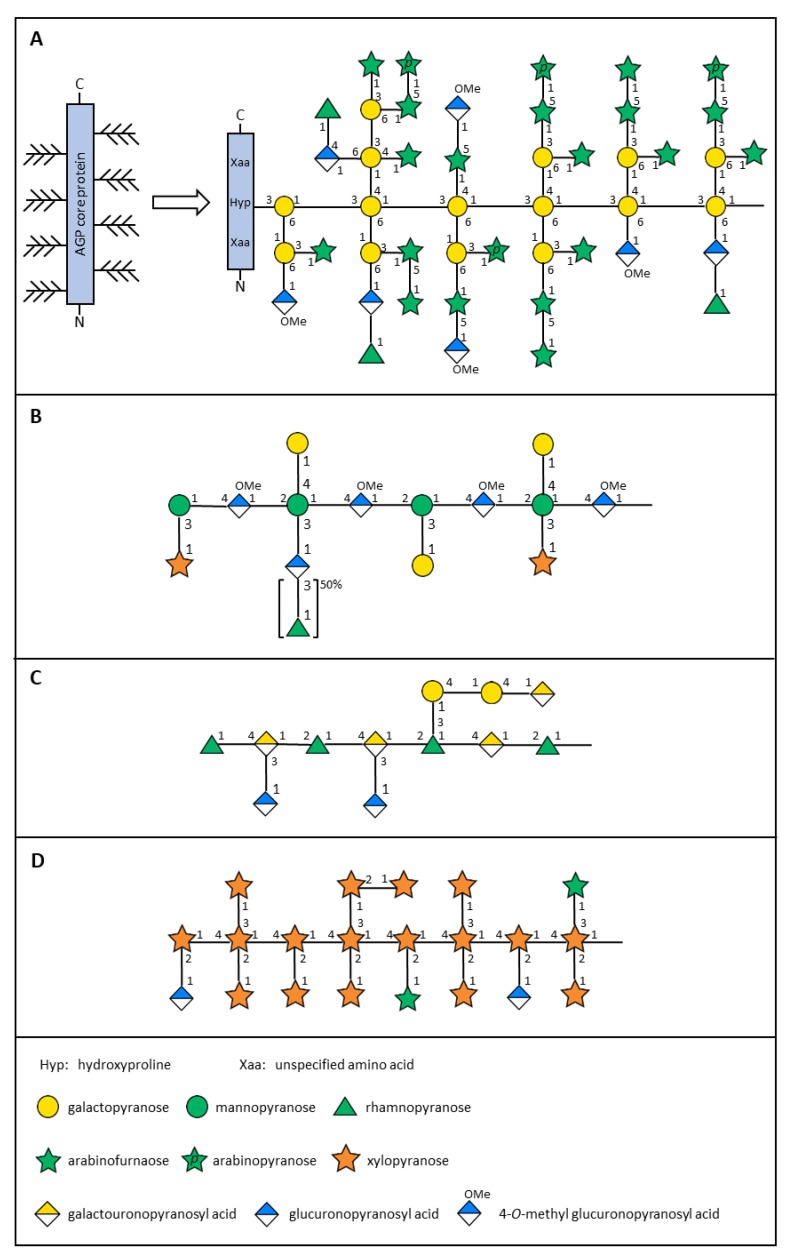
Schematic representations of possible structures for (**A**) puka gum, (**B**) mamaku polysaccharide, (**C**) houhere mucilage, and (**D**) harakeke mucilage.

Puka gum has structural features that are typical of classical AGPs: it comprises of about 2% protein that is rich in hydroxyproline and ~95% carbohydrate comprising mostly arabinose and galactose, together with smaller amounts of rhamnose and glucuronic acid [14]. Glycosyl linkage analysis and NMR spectroscopy shows that the gum has a highly branched backbone of β-1,3-linked galactopyranosyl residues, with side-chains containing arabinofuranosyl (Ara*f*) oligosaccharides terminated variously by Rha*p*, Ara*p*, Gal*p*, and Glc*p*A residues (Table 1). The general structure of puka gum (Figure 1A) is similar to that of the commercial AGP, gum arabic, but the molecular weight of puka gum (~4.5 × 10^6^ g/mol) is about seven times greater than that of gum arabic. Glycosyl linkage analysis and NMR spectroscopy shows minor differences in the side-chain oligosaccharides, with puka gum containing 5-linked Ara*f*, terminal Ara*p*, and terminal 4-*O*-methyl Glc*p*A that are not present in gum arabic; conversely gum arabic contains terminal Gal*p* that is absent from puka gum [14]. These differences present interesting properties and potential applications for puka gum.

Gum arabic is used as an emulsifier, stabilizer, and thickener in a range of foods, beverages, and confectionery. In one particular application, it is used as an emulsifier and stabilizer of flavor oils used in soft drinks. A recent study to test the potential of puka gum to act as an emulsifier for orange oil flavor emulsion concentrates showed that the smallest diameter oil droplets (~0.8 μm) were obtained using 10% puka gum and 15% weighted orange oil [18]. However, it was concluded that, due to beverage instability, puka gum is not an ideal emulsifier for beverage flavor emulsions, but has potential as an emulsifier in other food applications. A study of the coacervation of puka gum with whey protein isolate showed that puka gum could form complexes with whey protein isolate and, therefore, had potential for encapsulation applications, but with different functionality to gum arabic [19]. Puka gum has also recently been the subject of detailed molecular, rheological, and physicochemical analyses that may provide further insight in to the potential applications of this proteoglycan [20].

### 2.2. Mamaku (*Cyathea medullaris*)

Mamaku, or black tree-fern, is a tall tree fern that is distributed throughout the south-west Pacific. In New Zealand and Australia, the pith of the trunk and fronds was consumed as a source of carbohydrate [21]. The pith also contains a mucilage that was used for various medicinal purposes, both externally, for wounds, and internally, as a vermifuge and as a treatment for diarrhea [1].

A partially purified, high molecular weight extract of mamaku fronds that displayed complex rheological properties contained about 80% carbohydrate that was rich in uronic acid [22,23]. Subsequent detailed analysis of the starch free, purified polysaccharide showed that it was a glucuronomannan comprising a backbone of β-1,4-linked methylesterified glucopyranosyl uronic acid and α-1,2-linked mannopyranosyl residues, branched at *O*-3 of 45% and at both *O*-3 and *O*-4 of 53% of the mannopyranosyl residues (Table 1; Figure 1B). Glycosyl linkage analysis indicated that the side-chains contained short oligosaccharides terminated by mostly β-D-galactopyranosyl and β-D-xylopyranosyl residues [16]. Gum ghatti, a commercially available gum exudate from the bark of the tree *Anogeissus latifolia* (Combretaceae) has a backbone structure similar to that of mamaku polysaccharide, but is highly substituted with complex arabinogalactan type polysaccharide side-chains [24].

Mamaku polysaccharide exhibits unique rheological properties, including shear-thickening and extensional viscosity [22,23,25]. This solution behavior is distinct from gum ghatti and a glucuronomannan isolated from sesame leaves that demonstrate shear-thinning behavior [26,27]. The shear-thickening behavior of mamaku polysaccharide was shown to be cation and hydrogen-bonding dependent [28]. They proposed that there were two possible mechanisms for this shear-thickening behavior: ion-bridging between polysaccharide chains in the presence of multivalent cations and intermolecular hydrogen bonding. From the rheological studies possible food industry applications for mamaku polysaccharide have been proposed, including the control of dysphagia and increasing satiety by controlling stomach motility [25,29].

### 2.3. Houhere (*Hoheria populnea*)

Houhere (lacebark) is a member of the Malvaceae, which includes okra (*Abelmoschus esculentus*) [30], marshmallow (*Althaea officinalis*) [31], rose mallow (*Hibiscus moscheutos*) [32], and kola (*Cola cordifolia*) [33], which all produce polysaccharide mucilages that have traditional medicinal uses. Two other New Zealand members of the Malvaceae (*Hibiscus trionum* and *Entelea arborescens*) are also known to produce mucilages. The mucilage produced by soaking the inner bark of houhere in water was applied to burns and was taken internally for digestive and respiratory ailments [1]. Mucilage is also produced by leaves and is currently used as an ingredient in a range of elixirs [34].

Structural analysis of the mucilage extracted from leaves of houhere shows that it has the same rhamnogalacturonan I (RG-I) type backbone of →4]-α-D-Gal*p*A-[1→2]-α-L-Rha*p*-[1→ as mucilages from other members of the Malvaceae [17]. Glycosyl linkage analysis indicated the presence of various side-chain moieties, including single β-D-Glc*p*A residues attached at *O*-3 of the 4-Gal*p*A backbone residues, and α-D-1,4-linked galactopyranosyl oligosaccharides (Table 1; Figure 1C). Similar analysis of mucilage extracted from the inner bark of houhere showed a much simpler composition with only three major linkages, 2-Rha*p*, 3,4-Gal*p*A, and terminal Glc*p*A present (Sims, unpublished observation). Mucilages extracted from other members of the Malvaceae, including from the inner bark of *Grewia mollis* (grewia gum) [35] and the roots of *Hibiscus moscheutos* [32], show the same composition and structure. The gum from the leaves of *Entelea arborescens* (whau) is composed of D-GalA, L-Rha, D-Gal, and L-Ara [21] and, therefore, may have a structure similar to that of the mucilages of other members of the Malvaceae. 

The rheological properties of houhere mucilage showed typical shear-thinning behavior consistent with intermolecular entanglement, but interestingly they were largely unaffected by changes in pH [34]. This pH independent solution behavior contrasted with that of the structurally similar grewia gum and okra mucilage (a partially methylated and *O*-acetylated RG-I polymer with short galactose side-chains [30]), which both showed pH-dependent rheological properties [35,36]. 

### 2.4. Harakeke (*Phormium tenax* and *P. cookianum*)

Harakeke or NZ flax (family Asphodelaceae) has a long history of use as a source of fiber for a wide variety of applications including clothing, foot-ware, and a range of baskets for numerous uses. In contrast, post-European settlement NZ flax was primarily used for rope-making. Medicinally, a mucilage, which exudes from the leaf bases, was used by Māori to soothe burns and other wounds, and was taken as a treatment for diarrhea [1]. 

Fifty cultivars of NZ flax are recognized which includes varieties of both *P. tenax* and *P. cookianum*. The mucilage isolated from the leaf bases contains mostly xylose, arabinose, and glucuronic acid, with considerable variation observed in the proportions of xylose and arabinose [37]. Cultivars with low xylose (and consequently high arabinose) correspond to *P. cookianum* and hybrids, while those with higher xylose contents are *P. tenax*. Structural analysis of harakeke mucilage shows that it is a highly branched, high molecular weight glucuronoarabinoxylan [15,38]. More than half of the sugars (mostly xylose, arabinose, and glucuronic acid) are terminal residues, with high proportions of both singly and doubly branched β-D-xylopyranosyl residues (Table 1; Figure 1D). Similar, highly branched xylans, which contain high proportions of terminal Xyl*p*, have been isolated from the seeds of *Plantago* spp. [39,40,41]. Corn fiber gum, extracted as a by-product of the milling industry, is also a highly branched glucuronoarabinoxylan, but, in contrast to the mucilages from harakeke and *Plantago* spp., contains side-chains of mostly terminal Ara*f* and short oligosaccharides with terminal Gal*p* [42]. Both *Plantago* seed mucilages and corn fiber gum and have been suggested as potential viscosity modifiers and/or emulsifiers/emulsion stabilizers [42,43,44,45]. 

## 3. Fructans

**Figure 2:**
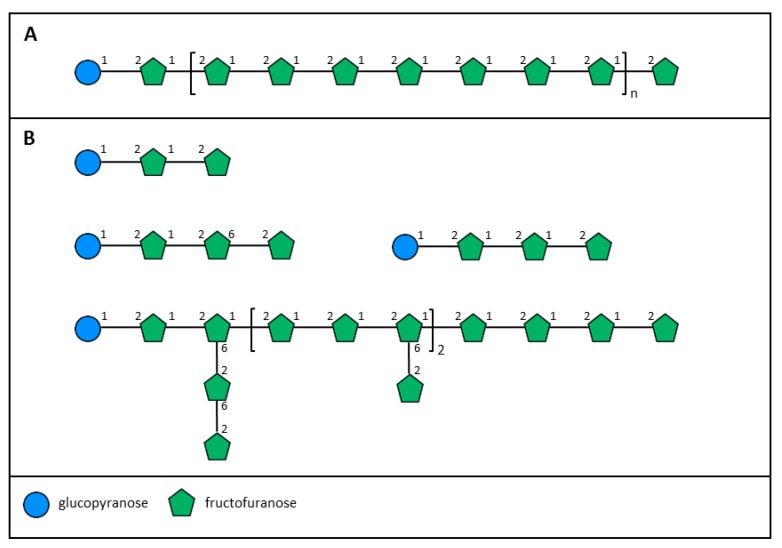
Schematic representations of (**A**) inulin and (**B**) trisaccharide, tetrasaccharides, and fructan from rengarenga.

Fructans are polymers of fructose that are synthesized from sucrose by the action of fructosyl transferases [46]. Inulin and fructo-oligosaccharides (Figure 2A), primarily from chicory roots, are used widely as food ingredients with beneficial health effects.

Fructans are commonly present in grasses and in the non-graminaceous order Asparagales. There are no publications on the specific occurrence of fructans in New Zealand grasses, but fructans are commonly found in the same genera as many endemic New Zealand grasses [47]. In the Asparagales, a fructan has been extracted from the roots of *Cordyline australis* (NZ cabbage tree); glycosyl linkage analysis and NMR spectroscopy showed that it was a branched fructan containing mostly 2,1-linked β-D-fructofuranosyl residues, with branching at *O*-6 of 15% of the fructosyl residues [48]. Subsequently, fructans have been shown to be present in other members of the Asparagales endemic to New Zealand. Branched fructans are found in underground parts of *Arthropodium cirratum* (rengarenga) and *Dianella nigra* (turutu, or New Zealand blueberry), and the flower stems of *Phormium* spp., while the bulbs of *Bulbinella hookeri* (Maori onion) contained inulin-type fructans [49,50]. Analysis of fructo-oligosaccharides isolated from rhizomes of *A. cirratum* showed that the major trisaccharide present was 1-kestotriose but that 6,1-kestotetraose was the most abundant tetrasaccharide; higher molecular weight fructan, with a degree of polymerisation greater than five sugars, showed the presence of both 2,1- and 2,6-linked β-D-fructofuranosyl residues, as well as 2,1,6-branched residues (Figure 2B) [50]. In contrast, the fructans from *B. hookeri* contained only 2,1-linked β-D-fructofuranosyl residues and were, thus, similar to inulin (Figure 2A).

## 4. Potential Utilization of New Zealand Polysaccharides 

Considerable research effort has been directed towards discovering new polysaccharides from New Zealand seaweeds, with desirable functional properties. This research resulted in the production of a pharmaceutical grade agar, although this has since lapsed due to the lack of a reliable supply of raw material [4]. The application of similar research suggests that there are also opportunities for commercial exploitation of new polysaccharides from New Zealand terrestrial plants in niche markets.

Gum arabic is used in cosmetics as an emulsifier and stabilizer in creams and lotions, to increase viscosity and provide a smooth feel on the skin [11]. Puka gum, which has a similar molecular structure, may have properties that make it suitable for such cosmetic applications. Indeed, Snowberry New Zealand Limited [51] cultivates puka trees for production of gum for use as an emollient and an antioxidant in skin-care products based on New Zealand ingredients. Harakeke mucilage is also an ingredient in skin-care products manufactured by several New Zealand companies, where it is used for its hydrating and moisturizing properties.

Polysaccharide gums are promising ingredients for foods designed for appetite control. The role of polysaccharides in appetite control and satiation has been reviewed by Fiszman and Varela [52]. To be effective in increasing satiety, polysaccharides need to display viscous behavior over a range of physicochemical conditions encountered in the gastrointestinal tract. The viscosity of mamaku polysaccharide and lacebark mucilage shows little variation over a wide pH range, and mamaku polysaccharide also shows a high tolerance of viscosity to changes in salt concentration [29,34] and, thus, may be effective in controlling satiety. Mamaku polysaccharide extract has been shown to delay gastric emptying and suppress appetite for up to 24 hours in rats, although further work is needed to make it suitable for use in humans, including reducing the starch and simple sugar content of the extract [53]. Houhere mucilage, on the other hand, may provide similar properties to mamaku polysaccharide, but does not contain starch. Also, extraction of houhere mucilage from leaves, rather than from bark, is a sustainable harvesting method that could be developed commercially.

Inulin and inulin derivatives, such as fructo-oligosaccharides (FOS), are well-established prebiotic ingredients used for their beneficial effects on gut microbiota populations [54,55]. Inulin is produced mostly from chicory roots, which contain 15%–20% fresh weight inulin [56], but is also extracted commercially from other plants including agave, a genus of fructan accumulating plants of the family Asparagaceae, grown commercially in Mexico. Another fructan-accumulating plant, yacon (*Smallanthus sonchifolius*), originating from South America, is grown in New Zealand and root powder and syrup are sold commercially. Our studies have shown that the yield of fructans from rengarenga (11% fresh weight), together with its of cultivation and past importance as a food source for Māori [57] make it a candidate for commercial production in New Zealand.

In conclusion, several polysaccharides with novel structures and properties have been isolated from New Zealand native plants. However, the high proportion of endemic New Zealand plants provides extensive opportunities for further discovery of new polysaccharides from unexplored species. Some of these polysaccharides are currently being used in niche “NZ Inc.” products and, with more research, show potential for utilization in a range of applications. 

## Figures and Tables

**Table 1:** Glycosyl linkage compositions of the gum and mucilage polysaccharides from NZ native plants.

		Composition (mol%)*^a^*			
Sugar	Linkage	Puka [14]	NZ flax [15]	Mamaku [16]	Houhere [17]
Rhamnose	terminal-*p*	9	-^*b*^	3	-
	2-*p*	-	-	-	22
	2,3-*p*				3
	2,4-*p*	-	-	-	8
Arabinose	terminal-*p*	8	-	2	-
	terminal-*f*	23	9	1	-
	3-*f*	7	-	-	-
	5-*f*	9	-	-	-
Xylose	terminal-*p*	-	33	9	-
	2-*p*	-	4	2	-
	4-*p*	-	2	3	-
	2,4-*p*	-	17	-	-
	2,3,4-*p*	-	16	-	-
Galactose	terminal-*p*	-	-	15	15
	3,6-*p*	19	-	1	-
	3,4,6-*p*	9	-	-	-
Mannose	2,3-*p*	-	-	9	-
	2,3,4-*p*	-	-	11	-
Galacturonic acid	terminal-*p*	-	-	-	12
	4-*p*	-	-	-	7
	3,4-*p*	-	-	-	14
Glucuronic acid	terminal-*p*	8	15	2	16
	3-*p*	-	-	6	-
	4-*p*	6	-	28	-
Other minor linkage		2	4	8	3

*^a^* values are the averages of duplicate analyses; ^*b*^-, not detected.
